# Supplementary material for: High-resolution genomic analysis to investigate the impact of the invasive brushtail possum (Trichosurus vulpecula) and other wildlife on microbial water quality assessments
Source: PLoS One. 2024 Jan 18;19(1):e0295529. doi: 10.1371/journal.pone.0295529 (PMC10796070; doi:10.1371/journal.pone.0295529)
Supplement: S2 File — (PDF) [file pone.0295529.s002.pdf]

Supplementary Figures S1-S6

High-resolution genomic analysis to investigate the impact of the invasive brushtail possum (*Trichosurus vulpecula*) and other wildlife on microbial water quality assessments

Marie Moinet[1], Lynn Rogers[1,2], Patrick Biggs[2], Jonathan Marshall[2], Richard Muirhead[3], Megan Devane[4], Rebecca Stott[5], and Adrian Cookson[1,2]

[1]AgResearch Hopkirk Research Institute, Palmerston North, New Zealand
[2]Massey University, *m*EpiLab, School of Veterinary Science, Palmerston North, New Zealand
[3]Massey University, School of Natural Sciences, Palmerston North, New Zealand
[4]Massey University, School of Mathematical and Computational Sciences, Palmerston North, New Zealand
[5]AgResearch Invermay, Mosgiel, New Zealand
[6]Institute of Environmental Science and Research Ltd. (ESR) Christchurch, New Zealand
[7]National Institute of Water and Atmospheric Research (NIWA) Hamilton, New Zealand

This Supplementary information PDF file contains:

S1 Fig: Venn diagram showing the overlap of gSTs between possums, other animals and environmental samples tested, at (A) the isolate level (63 different gSTs) or (B) the sample level (568 different gSTs). 2
S2 Fig: Distribution by sample type of the 20 most frequent ASVs (gSTs) identified by gnd metabarcoding. 2
S3 Fig: Metabarcoding sampling depth and observed richness (i.e. numbers of ASVs (gSTs)) 3
S4 Fig: DivNet individual alpha diversity estimates (Shannon index) 4
S5 Fig: DivNet estimates of beta diversity (Bray-Curtis distances) between pairs of sample types 4
S6 Fig: Minimal Spanning tree and subtree of 152 ST681 and 6 ST11707 genomes available in Enterobase as of 11 Jan 2023. 5

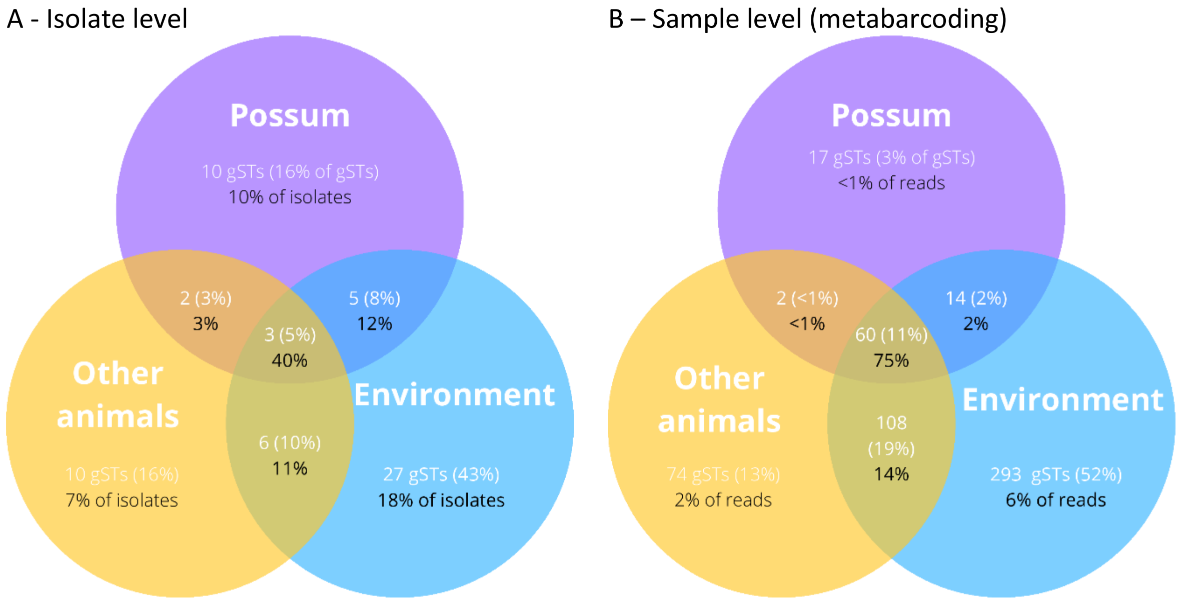

S1 Fig: Venn diagram showing the overlap of gSTs between possums, other animals and environmental samples tested, at (A) the isolate level (63 different gSTs) or (B) the sample level (568 different gSTs).

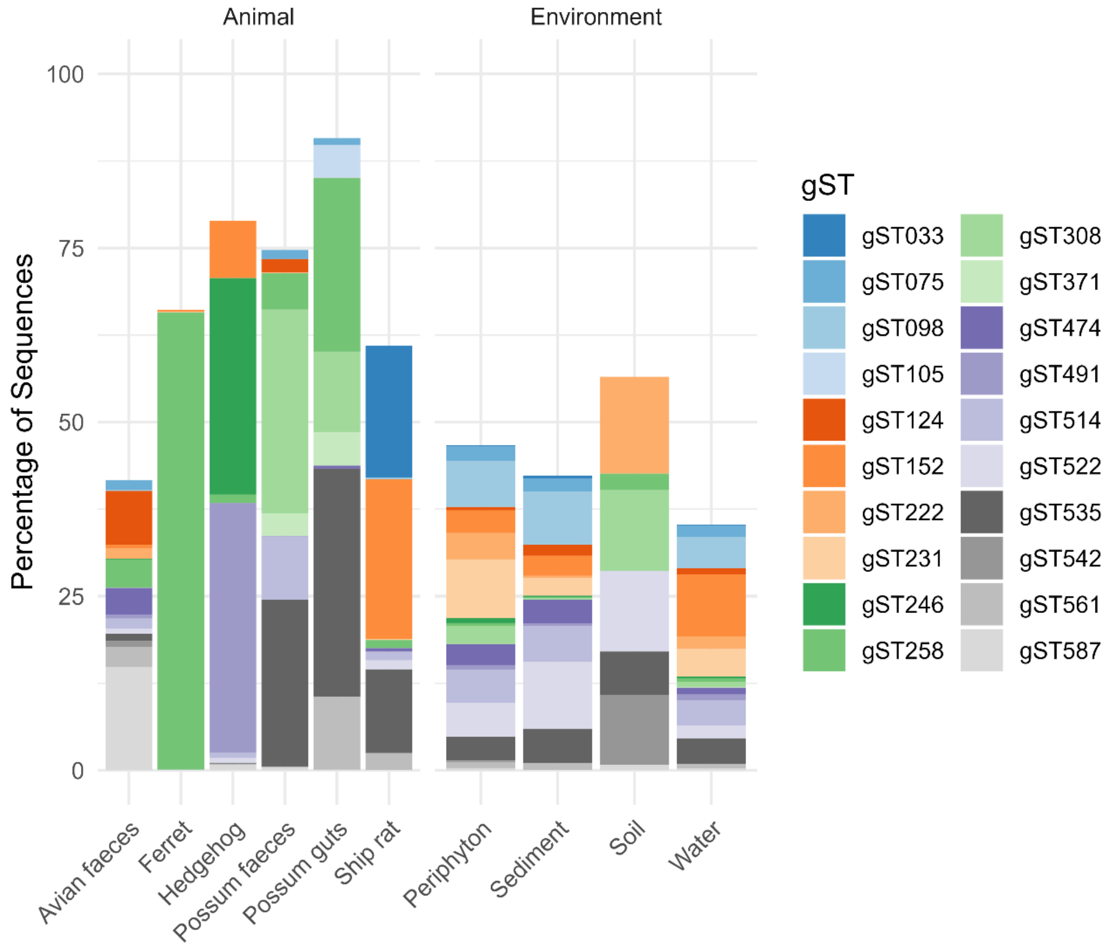

S2 Fig: Distribution by sample type of the 20 most frequent ASVs (gSTs) identified by gnd metabarcoding.

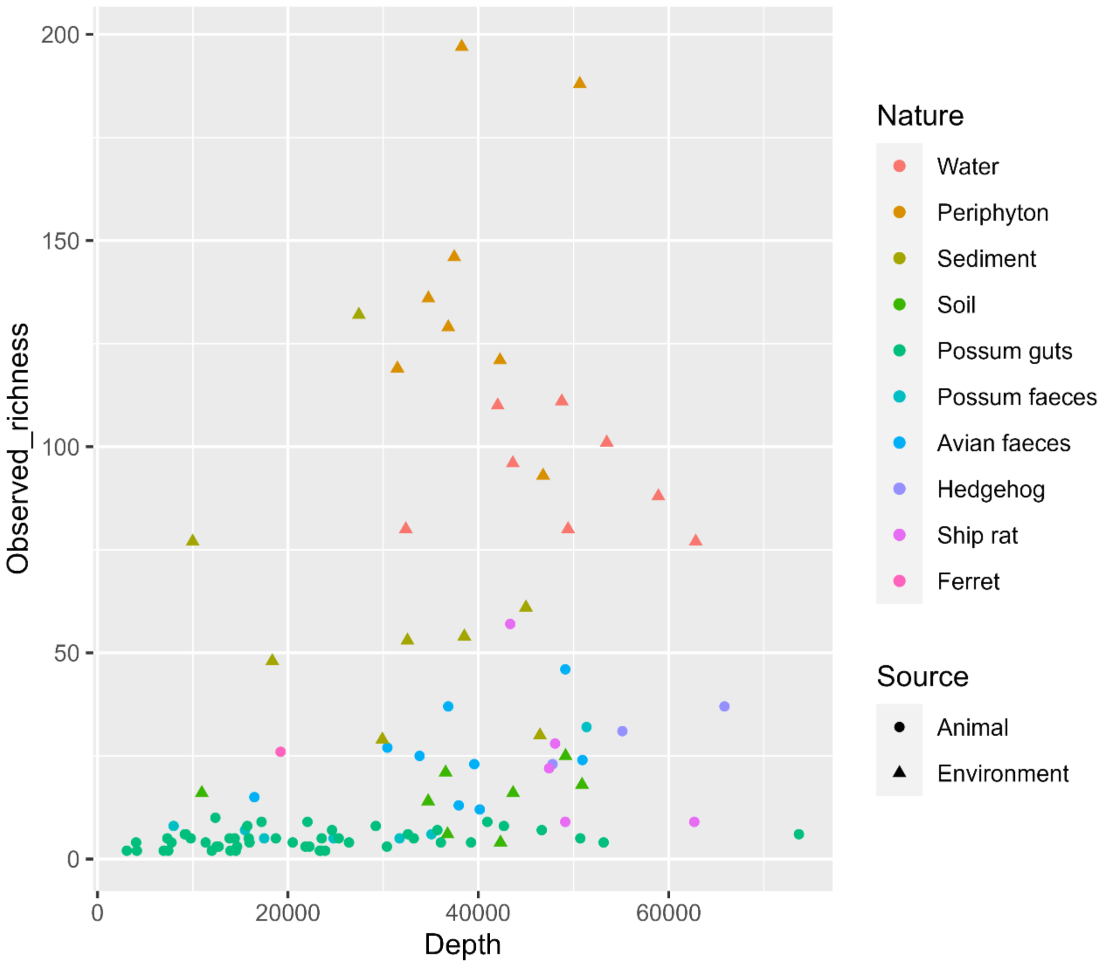

S3 Fig: Metabarcoding sampling depth and observed richness (i.e. numbers of ASVs (gSTs))

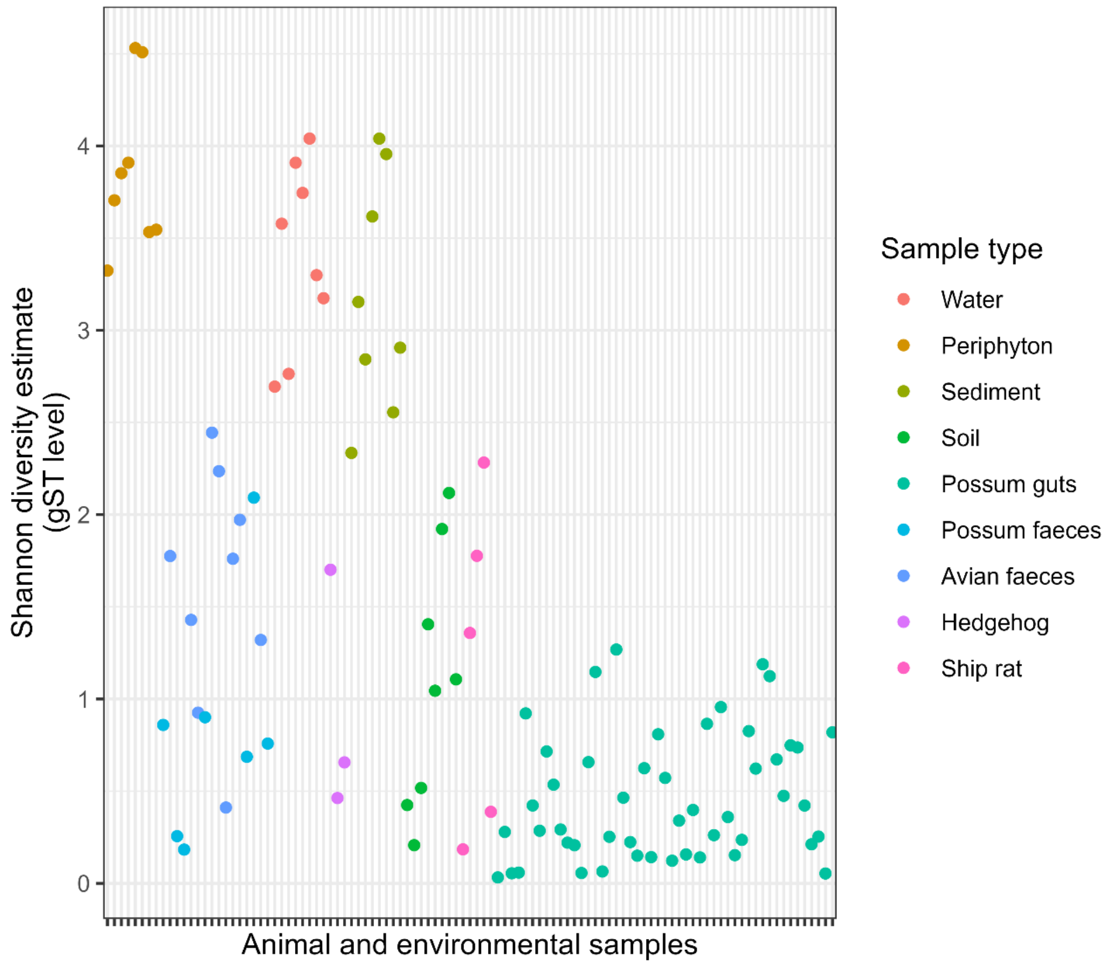

S4 Fig: DivNet individual alpha diversity estimates (Shannon index)

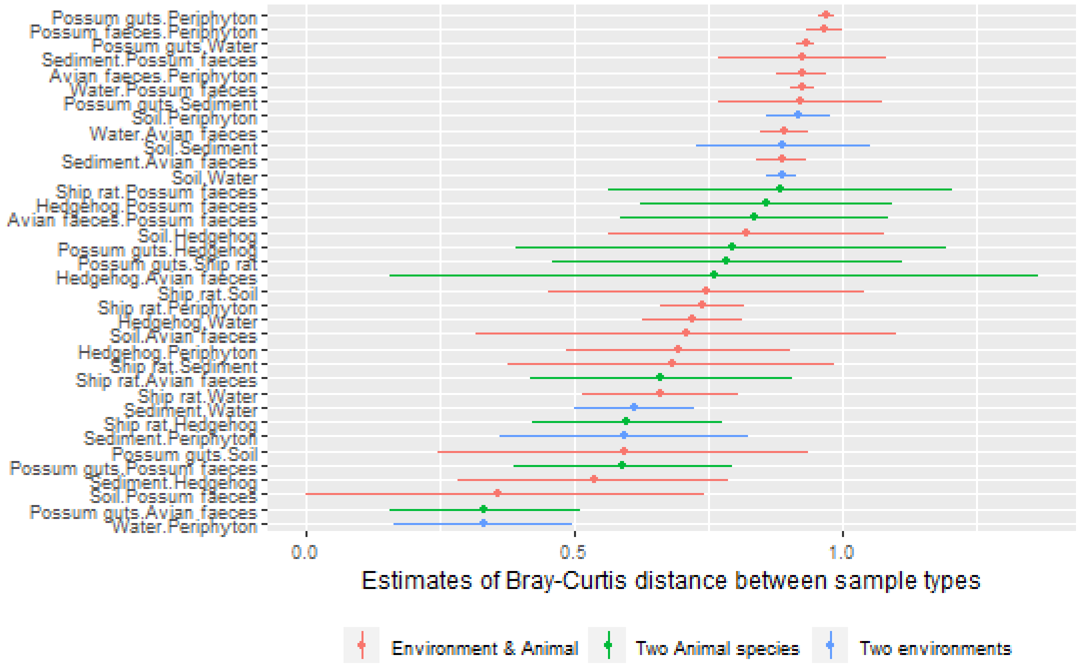

S5 Fig: DivNet estimates of beta diversity (Bray-Curtis distances) between pairs of sample types.

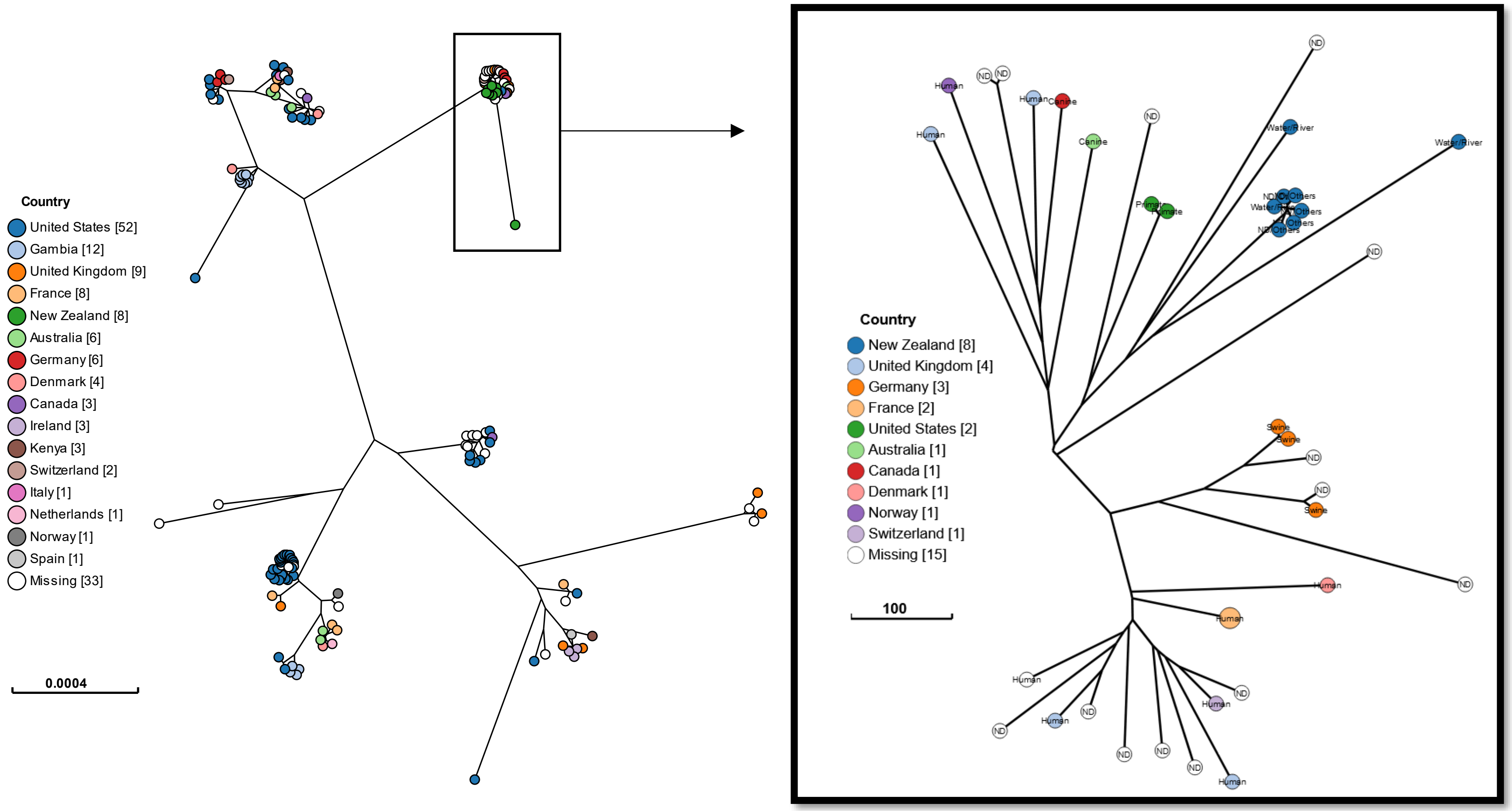

S6 Fig: Minimal Spanning tree and subtree of 152 ST681 and 6 ST11707 genomes available in Enterobase as of 11 Jan 2023. All 6 ST11707 available were isolated in New Zealand and appear clustered in the subtree. Tree built within Enterobase using the Grapetree module. The genomes from New Zealand are AGR3625 to AGR4170 (ref. 34) also appearing in Fig. 3.
