# Supplementary material for: High-resolution genomic analysis to investigate the impact of the invasive brushtail possum (Trichosurus vulpecula) and other wildlife on microbial water quality assessments
Source: PLoS One. 2024 Jan 18;19(1):e0295529. doi: 10.1371/journal.pone.0295529 (PMC10796070; doi:10.1371/journal.pone.0295529)
Supplement: Attachment — Submitted filename: ResponseToReviewers_PlosOne_fv.pdf [file pone.0295529.s003.pdf]

Adrian Cookson
AgResearch, Hopkirk Research Institute
Private Bag 11008,
Palmerston North 4442, NZ
+64 6-351 8681
Adrian.Cookson@agresearch.co.nz

Dr. Christopher Adenyo
Academic editor
Public Library of Science ONE
August 28, 2023

PONE-D-23-24161

Dear Dr. Adenyo,

We would like to express our gratitude to you and the reviewers for the insightful feedback provided on our manuscript titled "High-resolution genomic analysis to investigate the impact of the invasive brushtail possum (*Trichosurus vulpecula*) and other wildlife on water microbial quality assessments." We appreciate the time and effort invested in evaluating our work, and we have carefully considered each of the comments and suggestions offered. We have made the necessary revisions to address the concerns raised and believe that our revised manuscript now complies with PLOS ONE's requirements and standards. Below, we provide a detailed response to each of the comments and suggestions provided by the reviewers.

Sincerely,

Adrian Cookson
Senior Scientist, Food Systems Integrity team
AgResearch

Editor's requirements:

1. PLOS ONE's Style Requirements and File Naming: We have ensured that our manuscript has been formatted according to PLOS ONE's style requirements and that files are named appropriately.

2. Experiments Involving Animals (Methods Section): We have moved the mention of the method of sacrifice (kill-traps, i.e. animals were collected dead in the traps) in the sentence describing our ethical considerations in the choice of trap types, and we expanded the Methods section to include the sentence "As with those traps animals are killed instantly upon capture, methods of anesthesia and analgesia and efforts to alleviate suffering were not applicable."

3. Copyediting for Language, Spelling, and Grammar: We have conducted thorough copyediting to enhance the language usage, spelling, and grammar throughout the manuscript, thus ensuring its clarity and readability. We swapped two words around in the title and corrected a few typos, all visible in the version with the tracked changes.

4. Data Availability Statement and Accession Numbers: We have provided the relevant repository information for our data in the manuscript:

"WGS data of the 101 bacterial isolates and *gnd* metabarcoding sequences of the 106 animal and environmental samples have been deposited to the NCBI under BioProject number PRJNA987865 (BioSamples SAMN35994385 to 35994485 for WGS and

SAMN36426751 to 36426856 for metabarcoding) in the form of forward and reverse raw reads fastq files."

The links included in the text will become active once the article has been accepted. Furthermore, for reviewers to be able to see the data before acceptance, a confidential link to reviewers has been provided with the initial submission to access the BioProject.

5. Figure Copyright Compliance: We have re-created Figure 1 using QGIS and Creative Commons Attribution License (CC BY 4.0) background imagery from LINZ, which aligns with PLOS ONE's copyright guidelines. Attribution to the source has been added to the caption with appropriate links according to LINZ guidelines:

Background aerial imagery sourced from Toitū Te Whenua LINZ CC BY 4.0 Imagery Basemap contributors.

This image was checked through PACE as suggested, as the previous images had.

6. Reference List Review and Retracted Papers: We have thoroughly reviewed our reference list to ensure its completeness and correctness. We confirm that our reference list contains only current and relevant references, with no citations to retracted papers. No references have been added in our manuscript since the initial submission.

Reviewer #1:

(NB: the line numbers indicated below refer to the initial submission, not the amended manuscript)

1. **Line 62-63: Please insert "an". Considerable resources are spent in an attempt to control possum** The sentence has been revised as suggested. It now reads, " Considerable resources are spent in an attempt to control possum numbers [...]"
2. **Line 123: The "of" sentence should be changed to "as".** We are unsure what the reviewer means by "The "of" sentence should be changed to "as"." The only "of" present in line 123 cannot be replaced by "as". The sentence has been revised for clarity nonetheless. It now reads "The geometric mean of the Most Probable Number (MPN) of *E. coli*/100 mL of water was 317 (geometric standard deviation factor ×/÷ 1.11) at the Mākirikiri sampling point and 487 (×/÷ 1.51) at the Confluence."
3. **Line 252: Since alpha diversity is used to established the diversity within a sample, I am thinking that the statement "The difference in alpha diversity between water and other sample types was significant at the 0.05 level, except for soil (p = 0.789) and ship rats (p = 0.074, Table 2)" is not reflecting the actual meaning and should be rephrased. This is a suggestion for your consideration "The difference in the alpha diversity of water and other samples types was significant at 0.05 level, except for soil (p = 0.789) and ship rats (p = 0.074, Table 2)."** We appreciate the reviewer's suggestion, and the sentence has been rephrased as follows: "the difference in the alpha diversity of water and other sample types was significant at the 0.05 level, except for soil (p = 0.789) and ship rats (p = 0.074, Table 2)."
4. **Line 261: As you indicated in parenthesis some gSTs were previously found in E. marmotae an E. ruysiae and E. whittamii, I kindly want to find out if these works have been published? If yes kindly included the reference.** We apologize for any confusion. The isolates of *E. marmotae*, *E. ruysiae*, and *E. whittamii* mentioned were references to gSTs identified only in those species in the gndDb. The text has been

revised to clarify this point with mention of the publication, and now reads: “Out of 26 different gSTs described only in cryptic clades in the gndDb database [35], 17 were detected in the metabarcoding dataset, namely gST536, gST537, gST539 to gST546, gST548, gST587, gST591, gST593 (previously identified in isolates of *E. marmotae* in the gndDb), gST549, gST551 (previously identified in *E. ruysiae*), and gST614 (previously identified in *E. whittamii*).”

5. **Line 281: Please rephrase. I suggest you make it “two measurements exceeded 540 MPN”** The sentence has been rephrased as suggested: "two measurements exceeded 540 MPN."
6. **Line 290: Please the unit, I think you should state it as 6 possum/ha for better clarity.** The unit has been added for clarity. It now reads, "roughly over 6 possums/ha (49 possums from 8 ha)".
7. **Line 347: Please insert “that”. “Suggesting that these virulence factors may offer advantages for survival”** The sentence has been revised to include "that".
8. **Line 351: Please are you suggesting that ST681 and ST11707 are associated with gST535? If that is the case, I will suggest that you rephrase the sentence for better clarity. “Sequence types (ST681 and ST11707) associated with gST535 were not found in human cases notified between 2019 and 2021 in New Zealand, suggesting this strain has limited public health implications” for your consideration. Additionally, I would like the author to provide further explanation in their materials and method section how they determined the association between the ST and gST.** We apologize for any confusion. We have rephrased the sentence as follows: " Sequence types ST681 and ST11707, found in gST535 isolates in this study and previous work [34], were not found in human cases notified between 2019 and 2021 in New Zealand, suggesting this strain has limited public health implications." The word ‘association’ may not have been the proper term to describe the sequence types found in gST535 isolates and has been removed. We did not perform any specific analyses to determine an association between ST and gST and therefore did not modify the material and method section.
9. **Line 355:** The sentence has been updated to include "that": "indicated that New Zealand strains did not cluster with isolates recovered in other countries."

Reviewer #2:

1. **Abstract (L30-32): It would be good to include some more specific details of sharing between possums and environmental samples in the abstract. What proportion of environmental isolates were also detected in possums? What proportion of possum isolates were shared with other animals?** We appreciate the reviewer's suggestion and have added more specific details to the abstract regarding the proportion of gSTs shared: “Forty percent of isolates *gnd*-typed and 75% of reads obtained by metabarcoding had gSTs shared between possums, other animals, and the environment”. As this information was not present in the main text, we also added this information in the results section, as well as an extra Venn diagram offering more details in the supporting information.
2. **Results (L128): please provide a brief description of the culture based methods used.** A brief description of the culture-based methods used has been provided as requested. The sentence now reads: “A total of 420 isolates of presumptive *E. coli* were obtained from 105/106 samples (99.1%) following enrichment in EC broth and subculturing onto CHROMAgar ECC, an *E. coli* selective medium”.

3. **Results (L142): clarify that the 55% refers to the two gSTs combined.** The text has been clarified to indicate that the 55% refers to the combined proportion of the two gSTs. It now reads “29 isolates each, in total 55% of fecal and gut possum isolates *gnd*-typed”.
4. **Results (L146-149): So the most abundant gSTs in possums were not detected at high rates in the environment and other animals but were present. A version of this would be good to include in the abstract.** We apologize for any confusion. The two most abundant gSTs isolated from possums were gST535 and gST258. To clarify, gST535 was the second most frequent gST isolated in environmental samples. It was not clearly written in the text, and we have now added this information, but gST258 was the most abundant gST isolated from other animals. So, it wouldn’t be true to say that “the most abundant gSTs in possums were not detected at high rates in the environment and other animals but were present”. We want to emphasize that given the relatively small number of isolates *gnd*-typed from each environmental sample and the high alpha-diversity identified by metabarcoding in these samples, the resolution of the culture-based method is too low to offer a good picture of all gSTs present and make meaningful inference of what is/isn’t shared between sample types.
5. **Results (L170): why was the Clermont method of E. coli phylogenetic grouping not conducted on all isolates to give a more representative picture of the phylogroup distributions between the sample types? This could easily be done on the 420 isolates – well at least those uidA positive.** We appreciate the suggestion. The main rationale for using the *uidA* PCR was for the preliminary identification of cryptic clades vs. *E. coli*. When the study was designed, we decided to not use the phylogroup level (and hence the Clermont PCR), as information on the phylogroup does not provide enough power for making concrete conclusions on what is present in environmental vs. animal samples. We studied and compared the diversity of *Escherichia* populations between sample types at the gST level, offering a better resolution than phylogroups, and we confirmed the presence of clonal strains between sample types using the highest possible resolution, i.e., WGS. The information on phylogroups inferred *in silico* for the isolates that were submitted to WGS was presented as it is useful information, but this information is not central to answering the research question.
6. **Results (L172): How did these correspond to the gSTs?** The correspondence between phylogroups and gSTs has been further explained in the manuscript, bearing in mind other comments from reviewer #1 (cf. comment #8).
7. **Results (L250-251): Please present the statistical significance of these comparisons.** We acknowledge the potential for confusion in our original presentation. The statistical significance of the comparisons appeared in the following sentence. We moved the reference to tables and figures at the end of this second sentence and added a linking word at the start to avoid any misunderstanding: “Despite the higher number of reads obtained from possums compared to other sample types, the alpha diversity (Shannon index) was higher in periphyton, water and sediment than in animal sources including possums. Indeed, the difference in the alpha diversity of water and other sample types was significant at the 0.05 level, except for soil ($p = 0.789$) and ship rats ($p = 0.074$, Table 2, S3 Fig).”.
8. **Discussion (L273): Prevalence yes, but these gSTs did not appear to be at high abundance in the environmental samples according to this statement from the results: “The three most frequent gSTs were gST152 (five isolates, 7.8% of environmental 145 isolates), gST535 and gST587 (both four isolates, each 6.3% of environmental isolates respectively).”** Regarding the reviewer’s concern that the gSTs shared between animal and environmental samples did not appear to be at high abundance in the environmental samples, we wonder if the reviewer meant relative abundance rather than (absolute) abundance. As mentioned in the reviewer #2 point 4 of this letter, the high alpha-diversity in environmental samples means there is a big

denominator for the total number of gSTs identified in this type of sample, and hence a small relative abundance. In terms of absolute abundance, these gSTs were among the most frequent gSTs detected in the environment. We checked the ASV table in the metabarcoding dataset and the abundance of gSTs in terms of reads confirms this, with gST535 the 4th and 1st most abundant gST in environmental and other animal samples, respectively, and gST258 the 26th and 8th most abundant (out of 568 gSTs). We added a sentence to emphasize this point: "Despite a low relative abundance of those shared gSTs in environmental samples, linked to the high alpha-diversity in this type of samples, those shared gSTs were among the most frequently detected."

9. **Discussion (L298): suggest changing "confirm" to "show" or similar. At present, this study does not provide any evidence that removing possums would increase water quality. Could the authors do an analysis to assess this? If you assume all gST535 and maybe gST258 isolates in the water were from possums, then how much would the E.coli concentrations in the water be reduced if they were removed?** We have rephrased the sentence to indicate another study "would be needed to confirm" if sustained pest control programs can improve water quality. Given the enrichment step in our methodology, we think using our data to give a quantitative estimate of water quality improvement would remain speculative and a specific study design should be implemented to answer this question without bias.

10. **Discussion (L302): Which analysis showed that there was no difference in community composition between sample types? This seems surprising and I'd like to see a permanova test to confirm that as well as a PCoA of the Bray Curtis distances.** We apologize for any confusion. The analysis mentioned there refers to the test mentioned L560-562 in the methods section: "The null hypothesis of equal median measured relative abundance across sample types was tested with the *testBetaDiversity()* function from the *DivNet* package with a diagonal design matrix using a bootstrapped pseudo-F test (10,000 iterations)." and L253-257 in the results: "As regards to the beta diversity, we failed to reject the null hypothesis of equal median measured relative abundance across sample types at the 0.05 level (bootstrap p-value = 0.714). In other words, despite lower alpha diversities in animal compared to environmental samples, we did not detect a significant difference in measured beta diversity (Bray-Curtis distances) between sample types (S4 Fig)".

    We had done a PCA during the exploratory analysis of the data, not a PCoA. Both this PCA and the PCoA as well as the permanova test requested by reviewer #2 are presented below.

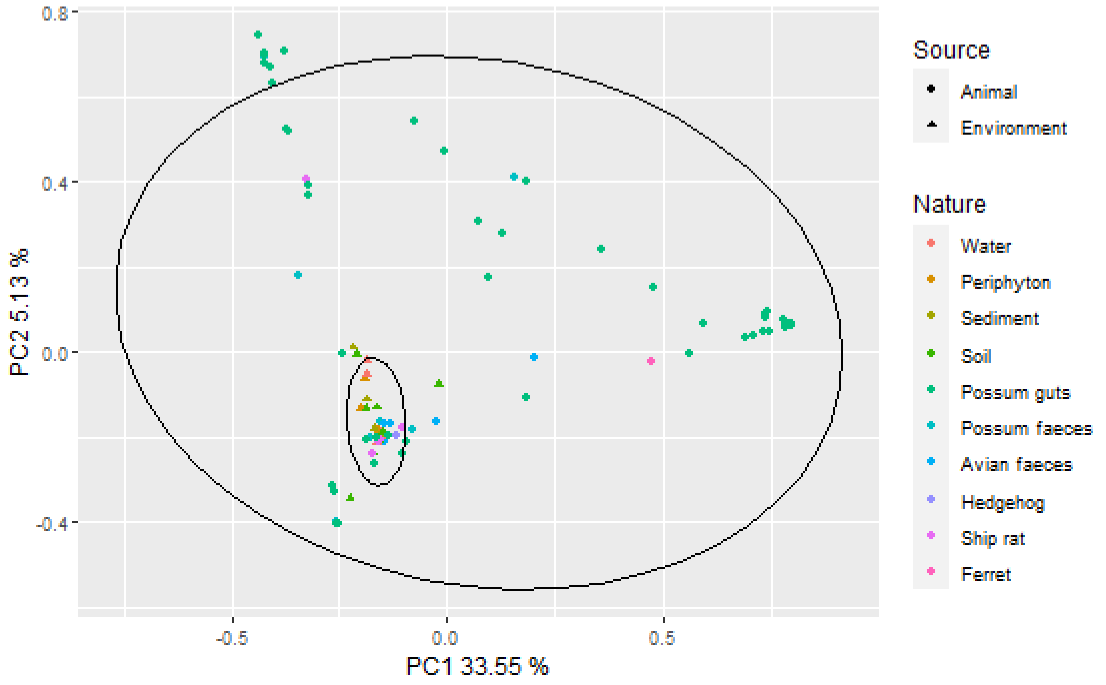

Figure 1: Principal Component Analysis of animal and environmental samples submitted to *gnd* metabarcoding (log transformed data).

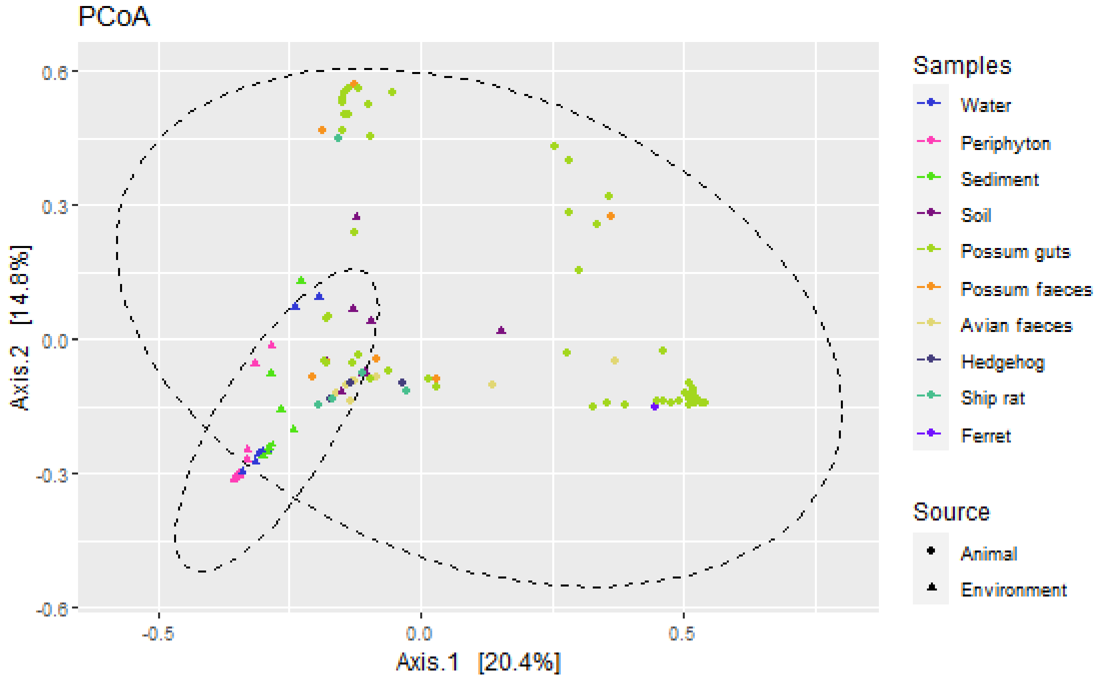

Figure 2: Principal Coordinates Analysis of animal and environmental samples submitted to *gnd* metabarcoding. A Lingoes transformation was used as there were negative eigenvalues.

As Reviewer #2 expected, the permanova detected significant beta diversity clustering of samples between sample types, but with non-homogenous dispersion between sample types:

```
Permutation test for adonis under reduced model
Terms added sequentially (first to last)
Permutation: free
```

```
Number of permutations: 999

adonis2(formula = distance_matrix ~ sample_data(ps)$Nature, add = TRUE)
                        Df SumOfSqs      R2      F Pr(>F)
sample_data(ps)$Nature   9   14.957 0.15735 1.9919  0.001 ***
Residual                96   80.097 0.84265
Total                  105   95.054 1.00000
---
Signif. codes:  0 '***' 0.001 '**' 0.01 '*' 0.05 '.' 0.1 ' ' 1
Homogeneity of multivariate dispersions

Call: betadisper(d = distance_matrix, group = sample_data(ps)$Nature)

No. of Positive Eigenvalues: 80
No. of Negative Eigenvalues: 25

Average distance to median:
        Water    Periphyton      Sediment          Soil   Possum guts
Possum faeces  Avian faeces      Hedgehog      Ship rat        Ferret
       0.4939        0.3769        0.5311        0.6478        0.5383
0.5670        0.6364        0.5575        0.6252        0.0000

Eigenvalues for PCoA axes:
(Showing 8 of 105 eigenvalues)
PCoA1 PCoA2 PCoA3 PCoA4 PCoA5 PCoA6 PCoA7 PCoA8
8.926 6.505 3.586 2.296 1.521 1.445 1.202 1.101

Permutation test for homogeneity of multivariate dispersions
Permutation: free
Number of permutations: 999

Response: Distances
          Df  Sum Sq  Mean Sq      F N.Perm Pr(>F)
Groups     9 0.74101 0.082335 3.7834    999  0.002 **
Residuals 96 2.08916 0.021762
---
Signif. codes:  0 '***' 0.001 '**' 0.01 '*' 0.05 '.' 0.1 ' ' 1
```

NB: When using the source (Environmental/Animal) as an independent factor rather than the sample type, the permanova also detected significant beta diversity clustering of samples between animal/environmental samples, but homogenous dispersion between animal/environmental samples, maybe because of the difference in dispersion between possums vs. other animal species and soil vs. other environmental samples. The betadisper plot is shown below:

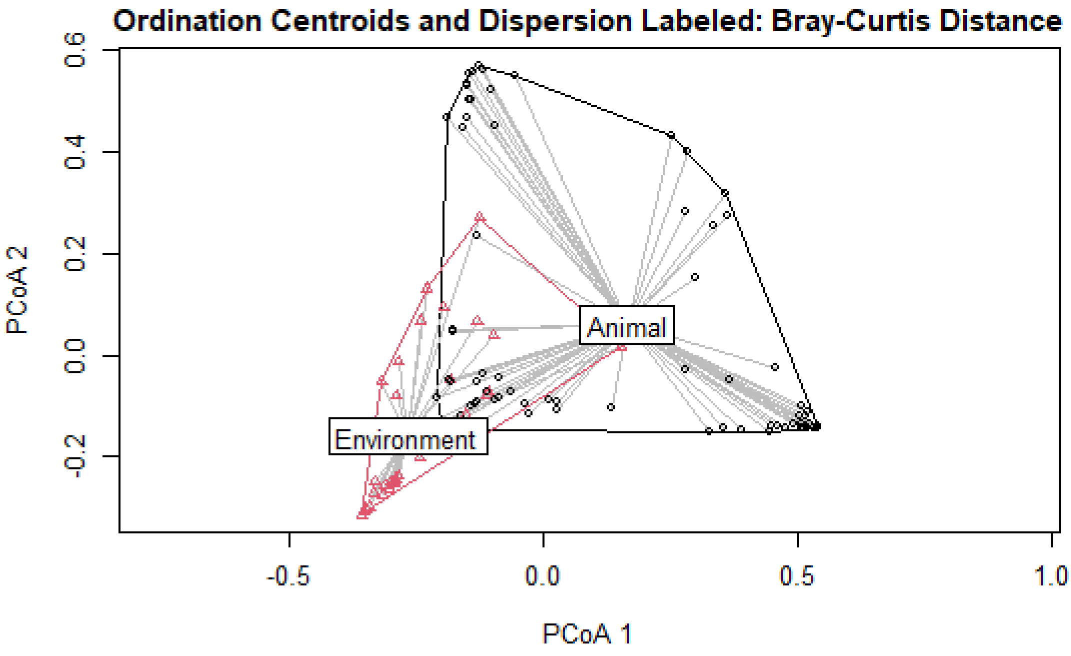

Figure 3: betadisper plot

The PCA and PCoA give similar results and identify a broad variation in distances among possums, scattered in a triangle (corresponding to the three main gSTs as shown in the biplot below). The variation in distances between samples composition is driven by possums, which can be explained by the presence of one of the dominating gSTs exclusive of other dominant gSTs in a given sample, coupled with a very low alpha-diversity in this species. It is unclear whether the dominance of one specific gST is real or artificially obtained due to the enrichment step allowing preferential growth of one strain. The first option is likely, as an analogous longitudinal study of commensal *E. coli* strains in an Australian population of mountain brushtail possums (*Trichosurus cunninghami*) also showed in that species, without pre-enrichment, a very low alpha-diversity and an average of 2.2 strains per possum, with changes in the main strain over sampling occasions [ref 40 in the manuscript]. A mention to this study has been added to the discussion.

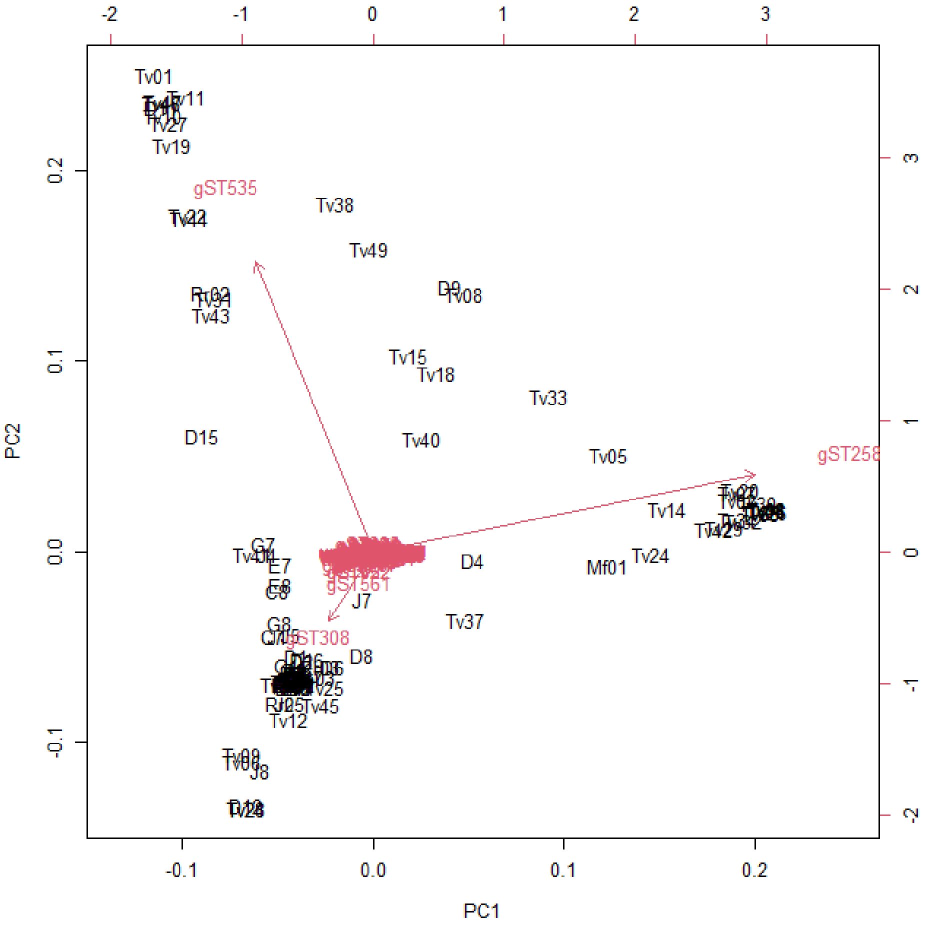

Figure 4: Biplot for the PCA of gSTs identified in possum (Tv), other wild species (Mf, Ee, Rr, C), and environmental samples (C, E, G, J)

The results are thus different from the tests on the DivNet model outputs. Please note that contrary to the PCoA and permanova, DivNet works by comparing habitats (or sample types), not individual samples.

Indeed, in both the PCoA/permanova and the DivNet approaches, the beta diversity is estimated using as metrics the Bray-Curtis distance (BCD), but there is a difference in how those distances are estimated and how the hypothesis of a difference is tested between groups.

In PCoA, BCD are calculated at the sample level, and a permanova (adonis2 in vegan) is then testing whether the 'average' community composition is similar between the different groups (in this case the different animal and environmental sample types).

With the DivNet approach, BCD are estimated at the sample type level (this means within a sample type, the Bray-Curtis distance will be zero), accounting for unobserved taxa. The testBetaDiversity() function then "uses output from DivNet() to estimate community centroids within groups defined by the groups argument [in this case the different animal and environmental sample types] and test a null hypothesis of equality of all group centroids against a general alternative" (https://rdrr.io/github/adw96/DivNet/man/testBetaDiversity.html).

As we were interested in comparing communities between the types of environments/animals species estimated thanks to available samples, rather than comparing communities between these samples, we consider it more appropriate and meaningful to describe the results based on the DivNet model (lack of evidence of a difference in estimated beta-diversity between habitats sampled), rather than the PCoA/permanova approach (difference in beta-diversity between observed samples of different types that may not be meaningful outside of our study). We also invite the reviewer to see the comment on this subject from the statistician who created DivNet at this link: https://github.com/adw96/DivNet/issues/13#issuecomment-411152644. She considers permanova inadequate for hypothesis-testing. For this reason, we answered the reviewer's query in this letter, but do not feel it would be suitable to present the PCoA/permanova results in the manuscript.

We have made mention of the use of the DivNet approach earlier in the results and discussion (and not just the methods section) to avoid any confusion, and have carefully rephrased the results ("did not detect a difference" rather than "there was no difference"). We have also modified the sentence in the discussion to more accurately discuss our results. The sentence now reads: "but we failed to find evidence for a difference in measured community composition between sample types."

11. **Discussion (L229-340): This is a good point and I think any abundance measures from this data should be interpreted with caution. However, I do think that they have some value where there are big differences between sample types, which might be worth discussing with the appropriate caveats.** Did the reviewer mean L339-340? We have modified the end of this paragraph to discuss with the appropriate caveats the main findings in terms of abundance of reads and isolates across sample types.

12. **Discussion (L353): But you just said monitoring is limited, so this statement can not be made with certainty.** There is indeed no certainty on the public health impact of gST535 and we used the verb "suggesting" in this sentence. We have modified the following sentence to underline this remains a hypothesis, it now reads: "[…] not found in human cases notified between 2019 and 2021 in New Zealand, suggesting this strain has limited public health implications. In support of this hypothesis, a phylogenetic tree […]"

13. **Discussion (L421): I think this is overstated. There needs to be evidence that possum gSTs are contributing to high E.coli loads in water before this statement can be made.** The statement has been adjusted to reflect the need for further evidence before making assertions about the impact pest control measures could have on water quality. The sentence now reads: "It is therefore realistic to hypothesize that the removal of invasive pests from the environment may also improve microbial water quality assessments in addition to enhancing endemic biodiversity."
